# Full genome SNP-based phylogenetic analysis reveals the origin and global spread of *Brucella melitensis*

**DOI:** 10.1186/s12864-015-1294-x

**Published:** 2015-02-18

**Authors:** Kim-Kee Tan, Yung-Chie Tan, Li-Yen Chang, Kok Wei Lee, Siti Sarah Nore, Wai-Yan Yee, Mohd Noor Mat Isa, Faizatul Lela Jafar, Chee-Choong Hoh, Sazaly AbuBakar

**Affiliations:** Tropical Infectious Diseases Research and Education Centre (TIDREC), University of Malaya, 50603 Kuala Lumpur, Malaysia; Department of Medical Microbiology, Faculty of Medicine, University of Malaya, 50603 Kuala Lumpur, Malaysia; Codon Genomics S/B, No 26, Jalan Dutamas 7, Taman Dutamas, Balakong, 43200 Seri Kembangan, Selangor Malaysia; Malaysia Genome Institute, Ministry of Science, Technology and Innovation, Jalan Bangi, 43000 Kajang, Selangor Malaysia

**Keywords:** *Brucella*, Phylogenetic, Genome, SNPs, Zoonotic, Bioterrorism

## Abstract

**Background:**

Brucellosis is an important zoonotic disease that affects both humans and animals. We sequenced the full genome and characterised the genetic diversity of two *Brucella melitensis* isolates from Malaysia and the Philippines. In addition, we performed a comparative whole-genome single nucleotide polymorphism (SNP) analysis of *B. melitensis* strains collected from around the world, to investigate the potential origin and the history of the global spread of *B. melitensis.*

**Results:**

Single sequencing runs of each genome resulted in draft genome sequences of MY1483/09 and Phil1136/12, which covered 99.85% and 99.92% of the complete genome sequences, respectively. The *B. melitensis* genome sequences, and two *B. abortus* strains used as the outgroup strains, yielded a total of 13,728 SNP sites. Phylogenetic analysis using whole-genome SNPs and geographical distribution of the isolates revealed spatial clustering of the *B. melitensis* isolates into five genotypes, I, II, III, IV and V. The Mediterranean strains, identified as genotype I, occupied the basal node of the phylogenetic tree, suggesting that *B. melitensis* may have originated from the Mediterranean regions. All of the Asian *B. melitensis* strains clustered into genotype II with the SEA strains, including the two isolates sequenced in this study, forming a distinct clade denoted here as genotype II*d*. Genotypes III, IV and V of *B. melitensis* demonstrated a restricted geographical distribution, with genotype III representing the African lineage, genotype IV representing the European lineage and genotype V representing the American lineage.

**Conclusion:**

We showed that SNPs retrieved from the *B. melitensis* draft full genomes were sufficient to resolve the interspecies relationships between *B. melitensis* strains and to discriminate between the vaccine and endemic strains*.* Phylogeographic reconstruction of the history of *B. melitensis* global spread at a finer scale by using whole-genome SNP analyses supported the origin of all *B. melitensis* strains from the Mediterranean region. The possible global distribution of *B. melitensis* following the ancient trade routes was also consistent with whole-genome SNP phylogeny. The whole genome SNP phylogenetics analysis, hence is a powerful tool for intraspecies discrimination of closely related species*.*

**Electronic supplementary material:**

The online version of this article (doi:10.1186/s12864-015-1294-x) contains supplementary material, which is available to authorized users.

## Background

*Brucella* spp. are listed as a category B potential biological warfare agent [1]. A low infectious dose of 100 to 1,000 organisms is sufficient to cause an infection. The mechanism of transmission, through aerosols or food chains, makes them easily transmissible to both humans and animals. Extensive studies have been done in the past exploring the potential of *Brucella* spp. as candidate biological weapon agents [2]. A biological warfare attack model built in 1997, based on an aerosol attack using *B. melitensis* in an urban population, was estimated could cause an economic loss of $477.7 million per 100,000 persons exposed [3]. Owing to the potentially insidious nature of a bioterrorism attack, the intentional release of any biological warfare agent could be difficult to detect. Tracking unusual strains in a particular region could serve as the first line of defence. Rapid detection of potential bioterrorism activities that usually starts with few indistinguishable cases from a natural outbreak, however, is difficult. It relies heavily on precise and sensitive methods, as well as the availability of information about the organism worldwide. Sporadic outbreaks of brucellosis occur worldwide, the probable geographical origin of the source of infection, however, is difficult to determine.

Current approaches to study *B. melitensis* genome are often limited to specifically targeted genes, for example, multi-locus variable-number tandem repeat (MLVA) analysis, multi-locus sequencing (MLS) and single nucleotide polymorphisms (SNPs) analysis are insensitive to detect new mutations from new clades and therefore, are not very useful [4,5]. Complete genome sequencing is the ideal. The methods for achieving it, however, were previously laborious, expensive and usually required time-consuming gap closing of genome assemblies. It was therefore only used for in-depth evolutionary analysis or screening of potentially useful SNPs or loci to develop assays such as the MLVA, MLS, multiple real time and gene sequencing [6]. With the development of next-generation sequencing (NGS) technologies, full bacterial genome sequencing has become highly accessible [4,7-11]. High quality draft bacterial genomes can be rapidly obtained. Unlike the taxonomically informative or canonical SNP-based approaches, whole-genome sequencing serves as a robust and unbiased method to resolve intraspecies relationships for closely related species such as *Brucella* spp. [12,13] and *Bacillus anthracis* [14,15].

To date, there are only a limited number of full genome sequences of *B. melitensis* isolates from different geographical locations available in publicly accessible databases. Only one draft full genome sequence of *B. melitensis* isolated from the Southeast Asia (SEA) region is available [16]. We identified and sequenced two human *B. melitensis* strains, MY1483/09 and Phil1136/12, which originated from Malaysia and the Philippines, respectively. We demonstrate here that a SNP-based phylogenetic analysis of the draft genome sequence obtained from a single sequencing run was sufficient to resolve the intraspecies genetic relationships of geographically distinct *B. melitensis* strains. In addition, whole-genome-based molecular epidemiology tracking allows the elucidation of the potential origins and spread of *B. melitensis.*

## Results

### General features

The full genome sequences of the *Brucella* isolates were generated from a single sequencing run using the 316 chips of the Life Technologies Ion Torrent PGM platform. The resulting reads were *de novo* assembled into two unclosed draft genomes, comprising 28 contigs with N50 of 249,915 bp and 251,380 bp for MY1483/09 and Phil1136/12, respectively. *B. melitensis* 16 M was used to assess the accuracy and effectiveness of the assembled contigs. The total contig sequence lengths for MY1483/09 and Phil1136/12 covered 99.85% (3,290,051 bp) and 99.92% (3,292,385 bp) when compared to the reference 16 M genome. The GC content of both isolates was 57.24%, which was similar to that of the 16 M strain (57.22%).

**Figure 1:**
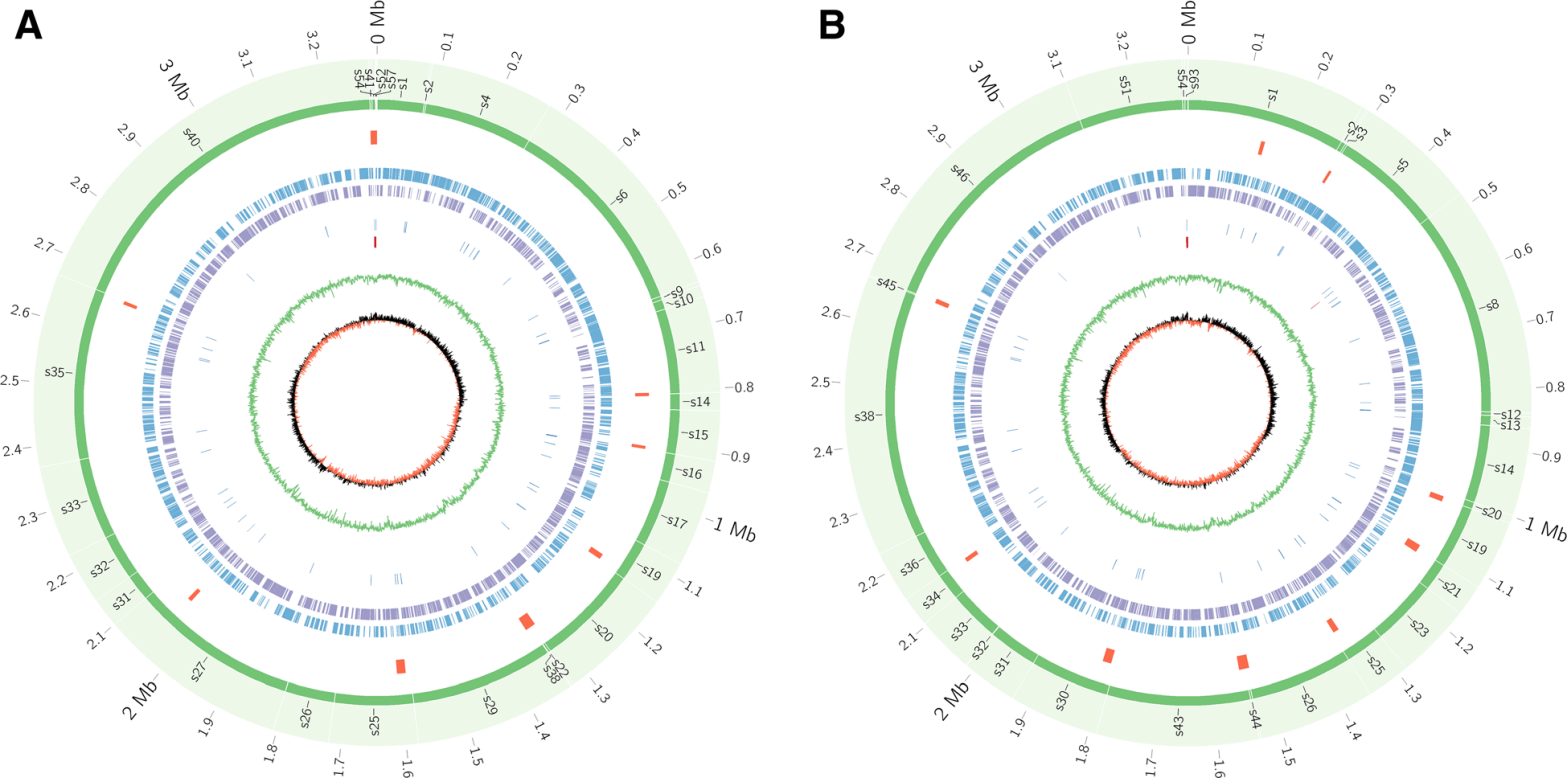
**Graphical circular map of the chromosome for (A) MY1483/09 and (B) Phil1136/12.** From the outside to the centre: scaffolds (green), genomic islands (red), putative genes in forward (blue), putative genes in reverse (purple), tRNA (dark blue), rRNA (dark red), GC plot (green) and GC skew (orange and black).

A total of 3,355 and 3,408 protein encoding genes were predicted and allocated in the chromosomes of the MY1483/09 and Phil1136/12 isolates, respectively, using GeneMarkS v4.10. The predicted functions of 2,920 genes of MY1483/09 (87% of total predicted genes) and 2,978 genes of Phil1136/12 (87% of total predicted genes) were obtained by searching against the NR, Swiss-Prot and KEGG databases. Three copies of rRNA in both isolates were identified using rRNAmmer v1.2. A total of 50 and 51 copies of tRNA were predicted using tRNAscan v1.23 in MY1483/09 and Phil1136/12, respectively (Figure 1A and B).

### Phylogenetic analysis

**Figure 2:**
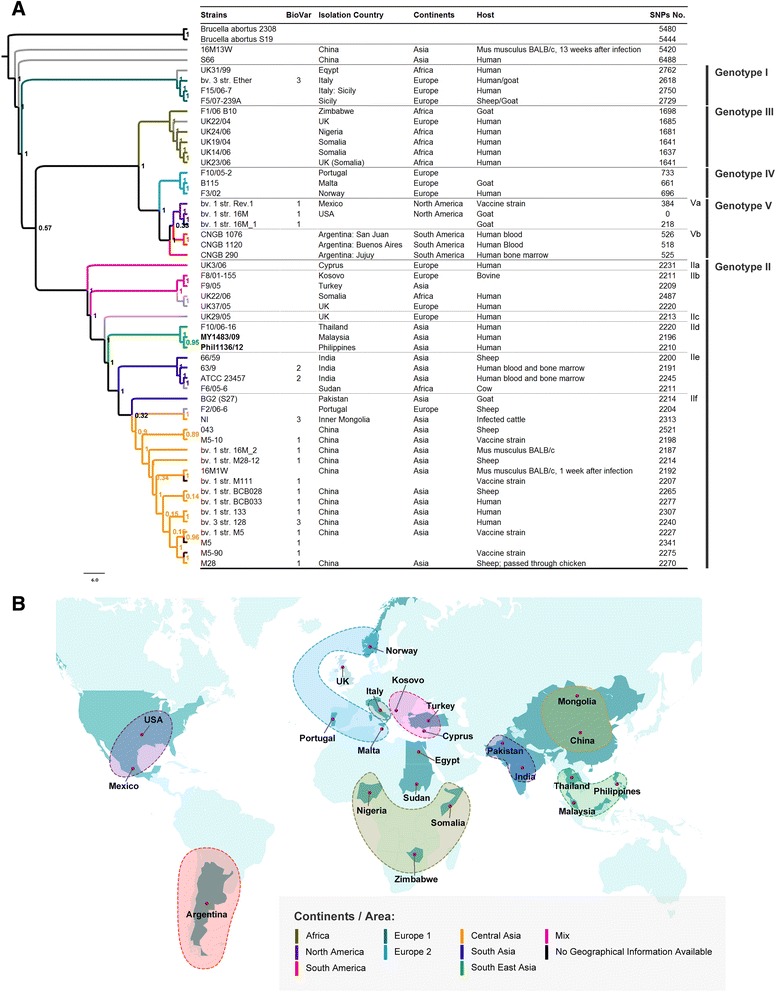
**Phylogeography of** ***B. melitensis*** **isolates collected globally. (A)** Phylogenetic tree of *B. melitensis* isolates. The phylogenetic tree was reconstructed using 13,728 polymorphic nucleotide sites and rooted with two *B. abortus* strains. **(B)** Geographical distribution of *B. melitensis* isolates collected globally.

The whole-genome-based SNP phylogenetic analysis was used to infer the relationships between *B. melitensis* strains collected from diverse geographical locations. We compared the draft genome sequence of the two isolates obtained in this study against 49 strains of *B. melitensis* (complete genome and draft genome) available in online databases at the time of the analysis. *B. melitensis* 16 M was used as the reference genome for sequence dataset, containing only the assembled sequences for the SNP discovery using SNPsFinder [17]. As a previous study indicated a close phylogenetic relationship between *B. melitensis* and *B. abortus* [4], the genomes of two *B. abortus* strains were selected as the outgroup in this analysis. The paralogous genes were removed from the analysis. A comparison of all *Brucella* strains, including the outgroup strains, with a mismatch cut-off of 8 bp, yielded a total of 13,728 polymorphic nucleotide sites (Additional file 1: Table S1). A phylogenetic tree was reconstructed using the identified SNPs shared among these genomes. The phylogenetic tree, together with the details of the isolates, including the geographical information, the host and the biovar were shown in Figure 2A. All of the *B. melitensis* strains, except 16M13W and S66, segregated into five major genotypes that corresponded spatially with the potential origins of the isolates. *B. melitensis* Ether (biovar 3) was basal to the *B. melitensis* clade. *B. melitensis* 16 M (biovar 1) and 63/9 (biovar 2) were separated after divergence from the Ether strain, demonstrating a similar tree topology that previously reported [4]. Incorporating the tree topology and the age of the *B. melitensis* lineage previously reported [4], we denoted *B. melitensis* Ether and its related strains as genotype I, *B. melitensis* 63/9 and its related strains as genotype II and *B. melitensis* 16 M and its related strain as genotype V. The *B. melitensis* genotypes III and IV were new genotypes that had not been reported, consisting mainly of recent isolates. Their genetic relationships with other *B. melitensis* strains have therefore not been well described [16]. The overall geographical distribution of the *B. melitensis* genotypes and clades is illustrated in Figure 2B.

Genotype I represented the most basal lineage of the *B. melitensis* strains. It comprised four out of the 49 *B. melitesis* strains used in the study. Based on the geographical origin of the strains, most of the genotype I *B. melitensis* strains were isolated from Italy (Ether, F15/06-7 and F5/07-239A) and one isolate, UK31/99 was collected from Egypt. The genotype I strains contained a number of SNPs, ranging from 2,618 to 2,762 in comparison to the *B. melitensis* 16 M. By conducting the SNP analysis, we identified 936 polymorphic sites that distinguished genotype I strains from other genotypes. A pairwise comparison of the genotype I isolates revealed the presence of 745 intragenotypic SNPs.

Genotype II comprised 30 out of the 49 *B. melitensis* strains used in the study. It represented the largest *B. melitensis* genotype, with isolates collected from diverse geographical locations, including African, European and Asian countries. It exhibited a ladder-like phylogram, suggesting a possible single introduction of genotype II strains into the region. The genotype II strains possessed SNP differences ranging from 2,187 to 2,521 in comparison to *B. melitensis* 16 M. Within genotype II, the isolates further segregated into several clades. A small clade, denoted group *a*, consisted of four *B. melitensis* strains that emerged from *B. melitensis* UK3/06 (Cyprus). The genotype II*b* strains were collected from distantly related geographical locations, with two of the strains having been collected from European regions, Kosovo and the United Kingdom (UK), one strain from Turkey (Asia) and one strain from Somalia (Africa). A pairwise comparison of the genomes revealed the presence of 295 SNPs within the II*b* strains. We identified 67 SNPs that were specific to *B. melitensis* genotype II*b.*

UK29/05, a *B. melitensis* strain collected from the UK, emerged from genotype II*b*. It occupied the internal node on the phylogenetic tree prior to the emergence of the Asian *B. melitensis* strains, herein denoted genotype II*c*. All Asian strains (except 16M13W and S66) diverged from genotype II*c*. Within the Asian lineage, we described the presence of three geographically distinct clades, SEA, India and China. The two isolates sequenced in the study clustered closely with the Thailand isolate F10/06-16, forming the SEA clade denoted group *d*. Within genotype II*d*, we identified 110 clade-specific SNPs. A pairwise comparison of the SEA clades revealed the presence of 15 SNPs within the SEA *B. melitensis* population.

The *B. melitensis* strains continued to diversify and split into two major clades (the Indian and China clades). They shared a common ancestor that was likely to have emerged from genotype II*d* (SEA clade). The isolates collected from India clustered with *B. melitensis* F6/05-6 collected from Sudan and formed the Indian clade (group *e*). Through the SNP analysis, we identified 51 clade-specific SNPs for genotype II*e* strains. A pairwise comparison of the Indian lineage revealed the presence of 56 SNPs. The China isolates clustered closely with three strains from Pakistan, Inner Mongolia and Portugal at a branch deep within the China clade, herein denoted group *f*. The laboratory strain, bv1 str. 16 M_2 (China 16 M), and its derivative, 16M1W, clustered within group *f*, but not with other *B. melitensis* 16 M strains (bv1 str. 16 M and bv1 str. 16 M_1) that were sequenced elsewhere. The 16M13W strain (another derivative of China 16 M) clustered neither in genotype II nor V, but in a group that did not belong to *B. melitensis*.

Genotypes III, IV and V emerged from the same common ancestral strains and demonstrated strong geographical clustering. Genotype III consisted of strains belonging to the African group, with the exception of UK22/04, which was collected from the UK. The isolates from genotype III had SNPs that numbered from 1,637 to 1,698 in comparison to *B. melitensis* 16 M. Based on the whole-genome SNP analysis, we identified 255 SNPs that were specific to genotype III strains. A pairwise comparison of genotype III strains revealed the presence of 1,388 intragenotypic SNPs. Genotype IV comprised isolates collected from Norway, Portugal and Malta (European clade). We identified 97 clade-specific SNPs that were shared among the genotype IV strains. A pairwise comparison of the genotype IV isolates showed 163 intragenotypic SNPs. Genotype V comprised the American strains collected from Argentina (CNGB1075, CNGB1120 and CNGB290), Mexico (Rev-1) and the USA (16 M strains except China 16 M). Within genotype V, the strains segregated into two groups, representing those from South America (Argentina) and those from North America (Mexico and USA). We identified 28 specific SNPs for the North American clades. However, we did not observe any specific SNPs for the South American clades.

Our analysis also included genome sequences of all of the vaccine strains of *B. melitensis* used worldwide. Rev-1 is a worldwide vaccine strain [18,19], whereas M5 and M111 are the vaccine strains currently used in China [8,10]. Rev-1 belonged to genotype V, grouping closely with 16 M and other isolates collected from Argentina. The M5 and M111 vaccine strains clustered with genotype II group *f*, the China clades. The M5 strain grouped closely with its parental strain, M28.

## Discussion

Anthropogenic activities allow infectious agents to adapt and be disseminated more efficiently to diverse geographical locations by their host. By tracking specific genome modifications, inference of the evolutionary history of the *B. melitensis* strains can be made. The *Brucella* spp. genome comprises two chromosomes without any additional extrachromosomal genetic elements. The horizontal transfer of genes, a common mechanism contributing to bacterial genome diversity, is limited in *Brucella* spp., perhaps due to its strict intracellular niche in the host [11]. Genome modifications, including mutations and genes rearrangements, in *Brucella* spp. therefore, are the exclusive heritable elements during the evolution of the bacteria [20]. The accumulation of mutations or acquisition of genome specific structural changes reflects the microevolution events that drive the evolutionary process, through genetic drift or bacterial adaption, upon introduction into a new geographical region or host. Among the *Brucella* spp., *B. melitensis* is the most virulent species for humans. Here, by incorporating a whole-genome SNP-based phylogenetic analysis and identifying the geographical origins of the isolates, we demonstrated a strong phylogenetic link in *B. melitensis* in relation to its distinctive geographical origins.

The presence of *Brucella* spp. in the ancient Herculaneum [21] and the high prevalence (17.4%) of brucellosis in Herculaneum adults [22] demonstrates the prolonged history and continuous circulation of *Brucella* spp. in the Mediterranean area. A high diversity of *B. melitensis* strains were collected from countries at the border of the Mediterranean Sea [23-25] and their basal location (genotype 1) on the phylogram suggests that *B. melitensis* could have originated from this region. This is further supported by the recent discovery of a 700 year-old *B. melitensis* genotype I strain from Sardinia [26]. Ancient and Old World transcontinental trading was an early form of globalisation that allowed the exchange of goods among the continents. The infectious agents that attached to humans or goods, such as livestock and its derivatives spread in a parallel manner [27]. Based on the phylogenetic tree drawn in this study using whole-genome SNPs, it was shown that the *B. melitensis* genotype I gave rise to two major branches, representing *B. melitensis* strains collected from the East (genotype II) and West (genotype III/IV/V). The strains were likely to have been distributed along the major ancient trade routes, e.g., the ancient Silk Road, the Trans-Eurasia exchange and the Columbian exchange. The transcontinental spread of *B. melitensis* could therefore have marked the beginning of the diversification of the *B. melitensis* strains. The global spread of *B. melitensis* may have occurred following these ancient trading routes, perhaps through infected goat and sheep or their milk derivatives.

*B. melitensis* genotype II had the most diverse geographical distribution after diverging from genotype I. Following the phylogenetic ladder pattern, the distribution of genotype II strains expanded from West (Cyprus) to East (China). Its ladder-like tree topology suggests that the genotype II strains descended from a single common ancestor that originated from the Mediterranean area (Cyprus origin?) to form the Asian clades, as almost all Asian strains of *B. melitensis* belonged to this group. It is noted that *B. melitensis* BG2 (S27), isolated from Pakistan, occupied the ancestral node of group *f* (China clade), suggesting that the *B. melitensis* strain currently circulating in China may has a Pakistani origin.

Genotypes III, IV and V of *B. melitensis* demonstrated a more restricted distribution. They shared a common ancestor, which most likely diversified and spread towards the southern region of the Mediterranean, and formed genotype III (African lineage). The strains that spread northwards could have further diversified and adapted geographically forming genotype IV (European lineage) and genotype V (American lineage). Compared with the American region, genotype IV of *B. melitensis* strains were more geographically diverse (Portugal, Malta and Norway). Unlike Portugal and Malta, Norway is officially a brucellosis-free country [28], with no *B. melitensis* having been detected in sheep or goat herds to date [29]. Therefore, the Norway strain, F3/02, most likely represents an imported case that probably originated in the northern part of the Mediterranean region. There were limited numbers of sequences available for analysis from this region. Thus, we cannot rule out the sampling bias that may have affected the assignment of genotype IV to the northern Mediterranean area.

The genotype V strains were most probably introduced from Europe into the American continent by infected animals several hundred years ago, where *B. melitensis* could have continued to diversify into two geographically specific sub-lineages, genotype Va (North American lineage) and Vb (South American lineage). Nevertheless, only two strains (three genome sequences) and three strains were used to designate genotypes Va and Vb, respectively. The two genotype Va strains, 16 M (USA origin) [30] and Rev-1 (Mexico origin; vaccine strain developed in USA laboratories) [31,32], however, may not reflect the currently circulating *B. melitensis* strain in the North American region. Similarly, all three *B. melitensis* strains used for the designation of genotype Vb were isolated from Argentina. Although the origin of the Argentinian strains in this study (native or imported) remains inconclusive, the continuing circulation of *B. melitensis* in Mexico and Argentina, where *B. melitensis* is the principle cause of brucellosis [33], suggests the existence of native *B. melitensis* in the American continent. With the current data, we demonstrated that genotype V of *B. melitensis* was only detected in the American region, supporting the geographical specificity of genotype V. More field *B. melitensis* strains from diverse geographical regions of the American continent, however, are needed to conclude the designation of genotype V.

The SEA region is believed to have been free of *B. melitensis* until recently, similar to Australia, New Zealand, Northern Europe, the US and Canada [34,35]*.* Recent epidemiological studies of livestock and *B. melitensis* associated with human brucellosis, however, have suggested that the bacteria could have been transmitted and maintained unknowingly in the goat and sheep populations [36-41]. These SEA *B. melitensis* strains have not been well studied. Genetic markers, such as IS711 used for species characterisation in previous studies, are not sufficient to delineate intraspecies relationships of *B. melitensis* [40,41]. Here, using a comprehensive whole-genome-based SNP approach, the two *B. melitensis* strains obtained in this study clustered closely with the Thailand *B. melitensis* strains (F10/06-16) and formed a new SEA clade. Deciphering the origin and source of the SEA *B. melitensis* strains is highly challenging, owing to the existence of both local and imported goat breeds in SEA countries. We identified 110 SEA clade-specific SNPs that differentiated the SEA clade from other global *B. melitensis* strains, suggesting a common ancestor of the SEA *B. melitensis* strains in this region. A previous study suggested that the persistence of *B. melitensis* in wildlife could serve as a reservoir for endemic outbreaks [42]. The high genetic similarity (15 polymorphic SNPs) of the three SEA *B. melitensis* strains*,* however, suggests that the spread of SEA strains was relatively recent. It is therefore unlikely that brucellosis was caused by autochthonous transmission from local breeds in the wild. Moreover, Malaysia, the Philippines and Thailand are not major exporters of goats and sheep in the region. Therefore, it is plausible that the *B. melitensis* in this region was introduced from a common source through the importation of goats and sheep from elsewhere. The bacterial strains became established and adapted to the local environment, leading to the spread of *B. melitensis* to the local goat and sheep populations.

In view of the persistent infection caused by the vaccine strains in animals [43,44], their possible involvement in human infection is worth investigating. Data concerning the endemic strains and the use of vaccines in local animal husbandry, however, have not been well documented. It is possible that the vaccine strains used in animals could intentionally be used to cause harm to humans. There are several veterinary vaccine *Brucella* spp. strains currently in use. We showed here, by using a whole-genome SNP-based phylogenetic tree, that we were able to resolve and discriminate between the vaccine strains and the endemic strains. This is particularly important to help identify possible sources of infection and to monitor the potential misuse of live attenuated vaccines.

Through the whole-genome SNP analysis, two previously assigned *B. melitensis* strains, S66 and 16M13W, segregated into a group that did not belong to *B. melitensis.* S66 was previously reported as a *B. melitensis* sequence type 8 strain [45], while 16M13W is the 13-week mice-adapted strain of China 16 M *B. melitensis* [9]. Clustering of the 16 M13W strain into a different group from its parental strain suggests that it may not have originated from China 16 M. Although both China 16 M and 16M1W were identified as *B. melitensis* strains, they were not grouped with other previously designated 16 M strains (bv1 str. 16 M and bv1 str. 16 M_1). In contrast, they grouped with other *B. melitensis* strains collected from China (genotype II), raising concern that the China 16 M may not be the same 16 M strain used worldwide.

To date, serological tests, such as the agglutination test, and isolation of bacteria from blood cultures remain the gold standard for the diagnosis of human brucellosis. The interpretation of the results from such assays, however can be difficult due to the serological cross-reaction of *Brucella* spp. with other bacteria [46,47]. In addition, differential identification of closely related *Brucella* spp. is not possible using a single serological test. The PCR-based genome sequence amplification method is a good alternative, as it allows for rapid identification of the bacteria. Differential identification of highly similar *Brucella* spp., however is only achievable with multiple gene sequence analyses, such as MLS and MLVA. With the limited candidate genes used for MLS and MLVA typing, the intraspecies relationships of closely related species of *B. melitensis* are difficult to resolve*.* In comparison to these subtyping approaches, the whole-genome SNP-based phylogenetic analysis presented here provides better resolution power to resolve the genetic relationships between the *B. melitensis* species.

## Conclusion

In summary, we have demonstrated that SNPs retrieved from the *B. melitensis* draft genomes were sufficient to resolve the interspecies relationships within *B. melitensis* strains*.* Our findings obtained using the genome SNP analyses revealed potential *B. melitensis* endemicity to the SEA region. The *B. melitensis* phylogeny presented here describes the relationships between the global *B. melitensis* strains and their evolutionary history. The whole-genome SNP-based approach provides an effective means of determining the native geographical origin of the pathogen and could serve as the basis for the reconstruction of the history of the global spread of *B. melitensis* at a finer scale*.* In addition, we have discriminated between the vaccine and endemic strains using the current whole-genome SNP approach. This will allow the rapid identification of pathogens and sensitive epidemiological follow-up in cases of release with intention to cause harm.

## Methods

### Bacterial strains

The *Brucella* strains MY1483/09 (GenBank: JN571438) [37] and Phil1136/12 were previously isolated from brucellosis patients admitted to hospitals in Kuala Lumpur. One patient had consumed unpasteurised cow’s milk in Malaysia (MY1483/09), while the other had drunk unpasteurised goat’s milk in the Philippines (Phil1136/12).

### Genomic DNA sample preparation

The bacteria were cultured in *Brucella* broth and incubated at 37°C for 72 hours. Genomic DNA was extracted from 5 mL of culture using the QIAamp DNA Mini Kit (Qiagen, Germany). All sample handling was performed in a BSL-3 biocontainment laboratory at the Tropical Infectious Diseases Research and Educational Centre (TIDREC), University of Malaya. The genomic DNA concentration was quantified using the Qubit® dsDNA BR Assay Kit (Invitrogen, Life Technologies, USA).

### Full genome sequencing

The genome library preparation was performed by using the Ion Xpress^TM^ Plus Fragment Library Kit (Life Technologies, USA) following the manufacturer’s protocol, 4471989 Rev E. The sheared DNA was separated using a 2% agarose gel (Promega, USA) and fragments that corresponded to 200 bp of a sequencing run (approximately 330 bp) were manually excised. The DNA was purified using the QIAquick Gel Extraction Kit (Qiagen, Germany). The library quality and concentration were assessed and determined using the 2100 Bioanalyzer (Agilent, USA) and High Sensitivity DNA Kit (Agilent, USA). The sequencing template was prepared using the Ion OneTouch^TM^ 200 Template Kit V2 DL (Life Technologies, USA) according to the manufacturer’s protocol. The Ion Sphere Particles were enriched using Dynabeads MyOne Streptavidin C1 beads (Invitrogen, Life Technologies, USA). The efficiency of the enrichment process was assessed using the Ion Sphere Quality Control Kit (Life Technologies, USA). Genome sequencing was undertaken using the Ion Torrent PGM sequencer (Life Technologies, USA).

### Genome assembly and annotation

The Ion Torrent generated reads were assembled against *B. melitensis* 16 M (GeneBank: NC003317 and GenBank: NC003318) using MIRA 3, as implemented in Torrent Suite V4.0.2. Assemblies of the two isolates were ordered and aligned based on that of *B. melitensis* 16 M by Mauve v2.3.1 [48]. Potential mis-assembly points identified from the alignment were verified by PCR and DNA sequencing using capillary electrophoresis. Scaffolds with mis-assembly points were manually broken and joined using Gap5 v1.2.14 [49]. The putative tRNA and rRNA were identified by tRNAscan v1.23 [50] and rRNAmmer v1.2 [51], respectively. The protein coding genes were predicted by GeneMarkS v4.10 [52]. The putative identities of these genes were annotated by running the Basic Local Alignment Search Tool (BLAST) [53] against the NCBI non-redundant, Swiss-Prot [54] and Kyoto Encyclopaedia of Genes and Genomes (KEGG) [55] databases.

### SNP discovery and phylogenetic construction

SNP divergence of all 53 genomes (Table 1, 2 draft genomes in this study, 5 complete and 44 drafts, publicly available genomes of *B. melitensis* and 2 genomes of *B. abortus* as outgroups) was discovered using the web-based programme SNPs Finder [17]. The SNPs were evaluated in homologous regions of 600 bp that shared sequence similarity of at least 99%. The repetitive/paralogous sequences in the alignment were eliminated by the algorithm in the SNPs Finder software [17] to reduce false positive SNP identification. The deduced SNP set was filtered by eliminating the SNPs that were close to each other at a distance of less than 8 bp, as previously reported [4], using in house scripts.

**Table 1:** ***Brucella*** **spp. strains used in the analysis in this study**

**No**	**Organism**	**Abbreviation**	**GenBank Assembly ID**
1	*Brucella melitensis* 043	043	GCA_000331655.1
2	*Brucella melitensis* 16 M13W	16 M13W	GCA_000250835.2
3	*Brucella melitensis* 16 M1W	16 M1W	GCA_000250815.2
4	*Brucella melitensis* 66/59	66/59	GCA_000366865.1
5	*Brucella melitensis* ATCC 23457	ATCC 23457	GCA_000022625.1
6	*Brucella melitensis* B115	B115	GCA_000365865.1
7	*Brucella melitensis* BG2 (S27)	BG2 (S27)	GCA_000370625.1
8	*Brucella melitensis* bv. 1 str. 133	bv. 1 str. 133	GCA_000298595.1
9	*Brucella melitensis* bv. 1 str. 16 M	bv. 1 str. 16 M	GCA_000007125.1
10	*Brucella melitensis* bv. 1 str. 16M_1	bv. 1 str. 16M_1	GCA_000160295.1
11	*Brucella melitensis* bv. 1 str. 16M_2	bv. 1 str. 16M_2	GCA_000250795.2
12	*Brucella melitensis* bv. 1 str. BCB028	bv. 1 str. BCB028	GCA_000292165.2
13	*Brucella melitensis* bv. 1 str. BCB033	bv. 1 str. BCB033	GCA_000292085.2
14	*Brucella melitensis* bv. 1 str. M111	bv. 1 str. M111	GCA_000209615.1
15	*Brucella melitensis* bv. 1 str. M28-12	bv. 1 str. M28-12	GCA_000209595.1
16	*Brucella melitensis* bv. 1 str. M5	bv. 1 str. M5	GCA_000348645.1
17	*Brucella melitensis* bv. 1 str. Rev.1	bv. 1 str. Rev.1	GCA_000158695.1
18	*Brucella melitensis* bv. 2 str. 63/9	63/9	GCA_000182235.1
19	*Brucella melitensis* bv. 3 str. 128	bv. 3 str. 128	GCA_000298615.1
20	*Brucella melitensis* bv. 3 str. Ether	bv. 3 str. Ether	GCA_000158735.1
21	*Brucella melitensis* CNGB 1076	CNGB 1076	GCA_000366625.1
22	*Brucella melitensis* CNGB 1120	CNGB 1120	GCA_000366885.1
23	*Brucella melitensis* CNGB 290	CNGB 290	GCA_000366905.1
24	*Brucella melitensis* F1/06 B10	F1/06 B10	GCA_000370645.1
25	*Brucella melitensis* F10/05-2	F10/05-2	GCA_000366925.1
26	*Brucella melitensis* F10/06-16	F10/06-16	GCA_000370665.1
27	*Brucella melitensis* F15/06-7	F15/06-7	GCA_000365845.1
28	*Brucella melitensis* F2/06-6	F2/06-6	GCA_000366945.1
29	*Brucella melitensis* F3/02	F3/02	GCA_000366965.1
30	*Brucella melitensis* F5/07-239A	F5/07-239A	GCA_000366985.1
31	*Brucella melitensis* F6/05-6	F6/05-6	GCA_000367005.1
32	*Brucella melitensis* F8/01-155	F8/01-155	GCA_000370685.1
33	*Brucella melitensis* F9/05	F9/05	GCA_000370705.1
34	*Brucella melitensis* M28	M28	GCA_000192725.1
35	*Brucella melitensis* M5	M5	GCA_000209575.1
36	*Brucella melitensis* M5-10	M5-10	GCA_000292065.1
37	*Brucella melitensis* M5-90	M5-90	GCA_000192885.1
38	*Brucella melitensis* MY1483/09	MY1483/09	In this study
39	*Brucella melitensis* NI	NI	GCA_000227645.1
40	*Brucella melitensis* Phil1136/12	Phil1136/12	In this study
41	*Brucella melitensis* S66	S66	GCA_000250775.2
42	*Brucella melitensis* UK14/06	UK14/06	GCA_000370725.1
43	*Brucella melitensis* UK19/04	UK19/04	GCA_000367045.1
44	*Brucella melitensis* UK22/04	UK22/04	GCA_000370745.1
45	*Brucella melitensis* UK22/06	UK22/06	GCA_000366585.1
46	*Brucella melitensis* UK23/06	UK23/06	GCA_000370765.1
47	*Brucella melitensis* Uk24/06	UK24/06	GCA_000370785.1
48	*Brucella melitensis* UK29/05	UK29/05	GCA_000370805.1
49	*Brucella melitensis* UK3/06	UK3/06	GCA_000370825.1
50	*Brucella melitensis* UK31/99	UK31/99	GCA_000370845.1
51	*Brucella melitensis* UK37/05	UK37/05	GCA_000370865.1
52	Outgroup *Brucella abortus* 2308	*Brucella abortus* 2308	GCA_000054005.1
53	Outgroup *Brucella abortus* S19	*Brucella abortus* S19	GCA_000018725.1

The phylogenetic relationships of the 53 genomes were constructed using MrBayes v3.2.1 [56]. Bayesian MCMC analysis was conducted by sampling across the entire general time reversible (GTR) model space. One million generations were run with a sampling frequency of 500 and diagnostics were calculated for every 5000 generations. A burn-in setting of 25% was used to discard the first 500 trees. Convergence was assessed manually with the standard deviation of split frequencies falling below 0.01. There was no obvious trend for the plot of the generation versus the log probability of the data (the log likelihood values) and the potential scale reduction factor (PSRF) was reasonably close to 1.0 for all parameters.

### Supporting data

The genome sequences generated in this study are available from the European Nucleotide Archive with accession numbers PRJEB7499 (http://www.ebi.ac.uk/ena/data/view/PRJEB7499) and PRJEB7504 (http://www.ebi.ac.uk/ena/data/view/PRJEB7504) for MY1483/09 and Phil1136/12, respectively.

## Additional file

Additional file 1: Table S1. List of 13,728 polymorphic nucleotide sites of *Brucella* spp. that used for phylogenetic tree reconstruction.
